# Usefulness of endotoxin activity assay for early diagnosis of sepsis

**DOI:** 10.1186/cc14129

**Published:** 2015-03-16

**Authors:** S Sekine, H Imaizumi, K Masumoto, H Uchino

**Affiliations:** 1 Tokyo Medical University, Tokyo, Japan

## Introduction

The purpose of this study was to evaluate the diagnostic and prognostic value of the endotoxin activity assay (EAA) of sepsis in patients with systemic inflammatory response syndrome (SIRS) and organ failure in ICU setting.

## Methods

In total, 76 patients with SIRS and organ failure or who were suspected of sepsis during critical care were included. According to the levels of EAA, all patients were classified into three groups (group L, EAA <0.4; group M, EAA ≥0.4 or EAA <0.6; group H, EAA ≥0.6). In order to evaluate the severity of illness, the Acute Physiology and Chronic Health Evaluation II (APACHE II) score, Sequential Organ Failure Assessment (SOFA) score and catecholamine (CA) index were recorded. Blood samples were obtained to measure EAA levels, inflammatory markers (procalcitonin, C-reactive protein and white blood cells), serum lactate level as an indicator of tissue hypoxia, and for blood culture. All patients were followed up for 6 months. APACHE II score, SOFA score, CA index, inflammatory markers, serum lactate levels and blood culture results were examined for diagnosis of sepsis, severe sepsis, septic shock and for prognosis of 30-day mortality. Each value was also compared with EAA levels.

## Results

Patient age was 69 ± 9.9 years (male: *n *= 48, female: *n *= 25). The total number of samples was 106 (group L/group M/group H: 35/35/36). Twenty-seven specimens were obtained from nonseptic patients and 83 specimens were obtained from septic patients. APACHE II score was highly correlated with SOFA score (*P *< 0.05). In group H, the APACHE II score was significantly higher (22.2 ± 0.8) than that in group M (18.4 ± 0.87) (*P *= 0.01). The SOFA score in group H was significantly higher (9.9 ± 0.5) than that in group M (7.5 ± 0.6) and group L (7.9 ± 0.6) (*P *= 0.006). EAA levels were significantly increased in septic patients (septic patients: 0.56 ±0.03, nonseptic patients: 0.42 ±0.05) (*P *= 0.011) and in the positive blood culture group (positive group: 0.66 ± 0.05, negative group: 0.48 ± 0.03) (*P *= 0.006). There was no relationship between EAA levels and other inflammation markers or 30-day mortality.

## Conclusion

In patients with suspected sepsis and positive blood culture, EAA levels were significantly increased and had strong correlation with severity of disease. This result suggests that EAA indicates the state of sepsis regardless of the possibility of infection in patients with SIRS with organ failure.

